# Ezetimibe blocks *Toxoplasma gondii*-, *Neospora caninum*- and *Besnoitia besnoiti*-tachyzoite infectivity and replication in primary bovine endothelial host cells

**DOI:** 10.1017/S0031182021000822

**Published:** 2021-08

**Authors:** Camilo Larrazabal, Liliana M. R. Silva, Carlos Hermosilla, Anja Taubert

**Affiliations:** Institute of Parasitology, Biomedical Research Center Seltersberg, Justus Liebig University Giessen, 35392 Giessen, Germany

**Keywords:** *Besnoitia besnoiti*, Ezetimibe, *Neospora caninum*, NPC1L1, *Toxoplasma gondii*

## Abstract

Coccidia are obligate apicomplexan parasites that affect humans and animals. In fast replicating species, *in vitro* merogony takes only 24–48 h. In this context, successful parasite proliferation requires nutrients and other building blocks. Coccidian parasites are auxotrophic for cholesterol, so they need to obtain this molecule from host cells. In humans, ezetimibe has been applied successfully as hypolipidaemic compound, since it reduces intestinal cholesterol absorption *via* blockage of Niemann−Pick C-1 like-1 protein (NPC1L1), a transmembrane protein expressed in enterocytes. To date, few data are available on its potential anti-parasitic effects in primary host cells infected with apicomplexan parasites of human and veterinary importance, such as *Toxoplasma gondii*, *Neospora caninum* and *Besnoitia besnoiti*. Current inhibition experiments show that ezetimibe effectively blocks *T. gondii*, *B. besnoiti* and *N. caninum* tachyzoite infectivity and replication in primary bovine endothelial host cells. Thus, 20 *μ*m ezetimibe blocked parasite proliferation by 73.1−99.2%, *via* marked reduction of the number of tachyzoites per meront, confirmed by 3D-holotomographic analyses. The effects were parasitostatic since withdrawal of the compound led to parasite recovery with resumed proliferation. Ezetimibe-glucuronide, the *in vivo* most effective metabolite, failed to affect parasite proliferation *in vitro*, thereby suggesting that ezetimibe effects might be NPC1L1-independent.

## Graphical abstract

**Figure:**
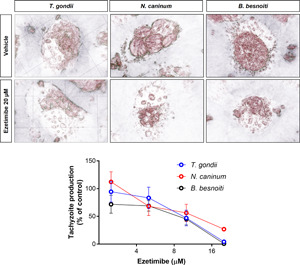

## Introduction

*Toxoplasma gondii*, *Neospora caninum* and *Besnoitia besnoiti* are cyst-forming species belonging to the Apicomplexa phylum, which consists of a large group of obligatory intracellular protozoan parasites that affect both humans and animals. Despite morphological similarities between coccidian species, host specificity and clinical consequences greatly differ among them. In this context, *T. gondii* is considered a major public health problem and an abortive agent especially in ovines (Benavides *et al*., 2017) and humans (Nayeri *et al*., 2020). The closely related coccidian parasite *N. caninum* is currently considered as a major cause of abortions in cattle (Reichel *et al*., 2013). In contrast, *B. besnoiti* causes bovine besnoitiosis, an emerging disease within Europe, which is characterized by massive alterations of skin and mucosas and also bull infertility (Alvarez-Garcia *et al*., 2013).

During the acute stage of infection, coccidian parasites undergo asexual replication within host cells. In this context, host endothelial cells have shown high permissiveness for tachyzoite infection and proliferation *in vivo* (Alvarez-Garcia *et al*., 2013; Konradt *et al*., 2016). Likewise, primary bovine endothelial cells have consistently been reported as suitable for *in vitro* replication of *T. gondii*, *N. caninum* and *B. besnoiti* (Taubert *et al*., 2006, 2016; Silva *et al*., 2019; Velásquez *et al*., 2019), allowing high tachyzoite proliferation rates in an experimental set up close to the *in vivo* scenario. During the fast proliferation phase, tachyzoites need significant amounts of nutrients for offspring development, which may be obtained from the host cell or newly synthesized. Specifically during coccidian replication high amounts of cholesterol are needed for new membrane biosynthesis (Coppens, 2013). Given that apicomplexan parasites are considered auxotrophic for cholesterol (Coppens, 2013), their replication within the parasitophorous vacuole (PV) highly depends on cholesterol supply by the host cell. In general, cellular cholesterol supply may be achieved either by enhancement of cellular endogenous *de novo* biosynthesis or by an increased cholesterol uptake from extracellular sources (Luo *et al*., 2020). In line, apicomplexan parasites can differentially exploit cholesterol sources depending on host cell type and parasite species. LDL internalization appears the main pathway for cholesterol uptake, and cholesterol esterification allows for storage in lipid-rich organelles (Luo *et al*., 2020). Recently, LDL-mediated cholesterol incorporation was described as pivotal, but not exclusive mechanism to fulfil cholesterol requirement during fast replicating coccidia proliferation (Nolan *et al*., 2015; Silva *et al*., 2019).

Based on pathophysiological consequences of human hyperlipidaemia, several pharmacological lipid-lowering compounds have been developed (Barter and Rye, 2016). Amongst these, ezetimibe is one of the most common hypolipidaemic drugs, which is capable of reducing intestinal cholesterol absorption by its interaction with Niemann−Pick C-1 like-1 protein (NPC1L1) in enterocytes (Davis *et al*., 2004; Garcia-Calvo *et al*., 2005). In detail, ezetimibe binds to NPC1L1, resulting in the blockage of NPC1L1 endocytosis into clathrin-coated vesicles and thereby diminishing cholesterol internalization into enterocytes (Ge *et al*., 2008; Wang *et al*., 2009). Despite that, the participation of other potential ezetimibe targets as the class B type 1 scavenger receptor (SR-BI) and the aminopeptidase N (CD13) have been linked to its hypolipidaemic effect (Kramer *et al*., 2005; Labonté *et al*., 2007). In general, the efficacy and safety of ezetimibe has been reported in mice and human studies (Bays *et al*., 2001; van Heek *et al*., 2001). As such, ezetimibe might represent a promising anti-parasitic drug candidate (Andrade-Neto *et al*., 2016). In line, ezetimibe treatments significantly reduced *Cryptosporidium parvum* growth in Caco-2 cells (Ehrenman *et al*., 2013). However, other evidences are incongruent: whilst ezetimibe reduced the parasite burden of *Leishmania amazonensis in vivo,* and diminished the *L. infantum* replication *in vitro* and *in vivo* (alone or in binary and ternary combination with miltefosine and itraconazole) (Andrade-Neto *et al*., 2016, 2021), this treatment did not affect *Plasmodium yoelii* parasitaemia in mice, while reduced the intraerythrocytic proliferation of *P. falciparum in vitro* (Kume *et al*., 2016; Hayakawa *et al*., 2021), thereby suggesting parasite-specific effects for this compound.

So far, no data are available on the impact of this drug on typical fast replicating coccidian parasites. Therefore, the aim of this study was to evaluate anti-parasitic efficacy of ezetimibe in *T. gondii, N. caninum* and *B. besnoiti*-infected primary bovine host endothelial cells.

## Materials and methods

### Host cell culture

Primary bovine umbilical vein endothelial cells (BUVEC) were isolated as described elsewhere (Taubert *et al*., 2006). BUVEC were cultured at 37°C and 5% CO_2_ atmosphere in modified endothelial cell growth medium (modECGM), by diluting ECGM medium (Promocell*^®^*) with M199 (Sigma-Aldrich) at a ratio of 1:3, supplemented with 500 U/mL penicillin (Sigma-Aldrich) and 50 *μ*g/mL streptomycin (Sigma-Aldrich) and 5% FCS (foetal calf serum; Biochrom). BUVEC of less than three passages were used in this study.

### Parasites

*Toxoplasma gondii* (strain RH) and *Neospora caninum* (strain NC-1) tachyzoites were cultivated *in vitro* as described elsewhere (Taubert *et al*., 2006; Velásquez *et al*., 2019), by maintaining them at several passages in permanent African green monkey kidney epithelial cells (MARC 145) in Dubelcco's modified eagle medium (DMEM) (Sigma-Aldrich). *Besnoitia besnoiti* (strain Bb Evora04) tachyzoite stages were propagated in Madin−Darby bovine kidney cells (MDBK) (Velásquez *et al*., 2020) in Roswell Park Memorial Institute (RPMI) medium (Sigma-Aldrich). All culture media were supplemented with 500 U/mL penicillin and 50 *μ*g/mL streptomycin and 5% foetal calf serum (FCS; Sigma-Aldrich). Infected and non-infected cells were cultured at 37°C and 5% CO_2_ atmosphere. Vital tachyzoites were collected from supernatants of infected host cells (800 × *g*; 5 min) and re-suspended in modECGM for further experiments.

For infection rate-related experiments, tachyzoites of each species were pre-incubated in 20 *μ*m ezetimibe for 1 h. After washing in modECGM (800 × *g*; 5 min), tachyzoites were used for infection experiments.

### Treatments of host cells and infections

BUVEC (*n* = 5) were seeded in 12-well plates (Sarstedt) pre-coated with fibronectin (1:400; Sigma-Aldrich). Ezetimibe (Cayman Chemical) and ezetimibe-glucuronide (Santa Cruz Biotechnology) stock solutions were prepared in dimethyl sulphoxide (DMSO; Sigma-Aldrich, 33 mm), diluted in modECGM at 2.5, 5, 10 and 20 *μ*m and administered to fully confluent cell monolayers 48 h before infection. ModECGM with DMSO (0.06%) served as vehicle control. Following pre-treatments, the medium was entirely removed and cells were infected with tachyzoites of *T. gondii*, *B. besnoiti* or *N. caninum* at a multiplicity of infection of 1:5 for 4 h under inhibitor-free conditions. Then, extracellular tachyzoites were removed and fresh medium with inhibitors was re-administered. At 4 h post infection (p. i.), phase-contrast images for infection rate estimation [(infected cells/total cells) × 100] were acquired by an inverted microscope (IX81, Olympus^®^) equipped with a digital camera (XM10, Olympus^®^). Tachyzoites present in cell culture supernatants were collected (800 × *g*; 5 min) at 48 h p. i. and counted in a Neubauer chamber.

Additionally, withdrawal experiments were carried out. Therefore, ezetimibe-containing medium was replaced by control medium at 24 h p. i., and parasite replication was estimated 24 h later (*n* = 5). Finally, further assays were performed to estimate the effect of ezetimibe over time as cells were treated as described above with a daily replacement of medium containing ezetimibe (20 *μ*m) at 24, 48 and 72 h p. i., and tachyzoite proliferation was observed at 48, 72 and 96 h p. i., respectively (*n* = 5).

### Live cell 3D holotomographic microscopy to illustrate parasite development

BUVEC were seeded into 35 mm tissue culture *μ*-dishes (Ibidi^®^) and cultured (37°C, 5% CO_2_) until confluence. Ezetimibe treatment (20 *μ*m) was performed as described above. Thereafter, *T. gondii*, *N. caninum* and *B. besnoiti* tachyzoites were used to infect cell layers (MOI = 3:1). At 24 h p. i., holotomographic images were obtained by using 3D Cell-Explorer-fluo microscope (Nanolive) equipped with a 60 × magnification (*λ* = 520 nm, sample exposure 0.2 mW/mm^2^) and a depth of field of 30 *μ*m. Images were analysed using STEVE software (Nanolive) to obtain refractive index (RI)-based z-stacks (Silva *et al*., 2019). Additionally, digital staining was applied according to the RI of intracellular tachyzoites. Finally, intracellular meront development was evaluated by counting intra-meront tachyzoites in at least six 3D holotomographic z-stacks of infected host cells ( = 50 cells per condition) in presence or absence of ezetimibe (20 *μ*m).

### RT-qPCR for relative quantification of NPC1L1 mRNA

BUVEC (*n* = 5) grown in 25 cm^2^ culture tissue flasks (Greiner Bio-One) were infected with *T. gondii*, *N. caninum* or *B. besnoiti* tachyzoites (MOI = 5:1). Infected- and non-infected host cells were processed for total RNA isolation at four different time points after infection (3, 6, 12, 24 h p. i.). Tissue samples from bovine small intestine obtained at a local slaughterhouse were used as positive controls for NPC1L1. For total RNA isolation, the RNeasy kit (Qiagen) was used according to the manufacturer's instructions. Total RNAs were stored at −80°C until further use. In order to remove any genomic DNA leftover, DNA digestion step was performed. Therefore, 1 *μ*g of total RNA was treated with 10 U DNase I (Thermo Scientific) in 1× DNase reaction buffer (37°C, 30 min). DNase was inactivated by heating the samples (65°C, 10 min). The efficiency of genomic DNA digestion was confirmed by no-RT-controls in each RT-qPCR experiment. cDNA synthesis was performed using the SuperScript IV (Invitrogen^TM^) according to the manufacturer's instructions. Briefly, for first-strand cDNA synthesis, 1 *μ*g of DNase treated total RNA was added to 0.5 *μ*L of 50 *μ*m oligo(dt), 1 *μ*L of 50 ng/*μ*L random hexamer primer, 1 *μ*L of 10 mm dNTP mix in a total volume of 10 *μ*L. Thereafter, the samples were incubated at 65°C for 5 min and then immediately cooled on ice. Additionally, 4 *μ*L of 5× SSIV buffer, 1 *μ*L 0.1 m DTT, 1 *μ*L RNAse free H_2_O and 0.5 *μ*L SuperScript IV enzyme were added obtaining a total volume of 20 *μ*L. The samples were incubated at 23°C for 10 min followed by 50°C for 10 min and an 80°C inactivation step for 10 min.

Probes were labelled at the 5′-end with a reporter dye FAM (6-carboxyfluorescein) and at the 3′-end with the quencher dye TAMRA (6-carboxytetramethyl-rhodamine). bNPC1L1 primer sequences were designed as follows: *Bos taurus* NPC1L1 forward 5′- CTTCCCTGATATGTCTTAC −3′; reverse 5′- GACCAGAGATATAAAGGC-3′ probe AGCCAGTCAATGAAGTCGTCCA. qPCR amplification was performed on a Rotor-Gene Q Thermocycler (Qiagen) in duplicates in a 10 *μ*L total volume containing 400 nm forward and reverse primers, 200 nm probe, 10 ng cDNA and 5 *μ*L 2× PerfeCTa qPCR FastMix (Quanta Biosciences). The reaction conditions were as follows: 95°C for 10 min, 40 cycles at 95°C for 10 s, 60°C for 15 s and 72°C for 30 s. No-template controls and no-RT reactions were included in each experiment. As reference gene GAPDH was used as previously reported (Taubert *et al*., 2006; Hamid *et al*., 2014; Hamid *et al*., 2015).

### Viability assessment

For experiments on parasite viability, 5 × 10^5^ tachyzoites of each parasite species were treated for 1 h with vehicle (DMSO 0.06%) or ezetimibe (20 *μ*m) (37°C, 5% CO_2_). Thereafter, viability of tachyzoites was determined by the trypan blue (Sigma-Aldrich^®^) exclusion staining assay as described elsewhere (Cervantes-Valencia *et al*., 2019). Non-stained parasites were considered as viable. Additionally, cell viability after compound treatments was assessed by the colorimetric XTT test (Promega^®^) according to the manufacturer instructions. Briefly, BUVEC seeded in 96-well plate (Greiner) were incubated with DMSO, ezetimibe or ezetimibe-glucuronide (both 20 *μ*m) in a total volume of 50 *μ*L for 72 h. Thereafter, 50 *μ*L of XTT working solution was added, and samples were incubated for 4 h (37°C, 5% CO_2_ atmosphere). The resulting formazan product was estimated *via* optical density (OD) measurements at 590 nm and reference filter 620-nm wavelength using Varioskan^TM^ Flash Multimode Reader (Thermo Scientific).

### Statistical analysis

For statistical analyses, the statistical software GraphPad^®^ Prism 8 (version 8.4.3.) was used. Data description was performed by presenting arithmetic mean ± standard deviation. In addition, the non-parametric statistical test Mann−Whitney for comparison of two experimental conditions was applied. In cases of three or more conditions, Kruskal−Wallis test was used. Whenever global comparison by Kruskal−Wallis test indicated significance, *post hoc* multiple comparison tests were carried out by Dunn tests to compare test with control conditions. Outcomes of statistical tests were considered to indicate significant differences when *P* ⩽ 0.05 (significance level).

## Results

### Ezetimibe treatments effectively block *T. gondii, N. caninum* and *B. besnoiti* tachyzoite proliferation

**Fig. 1.**
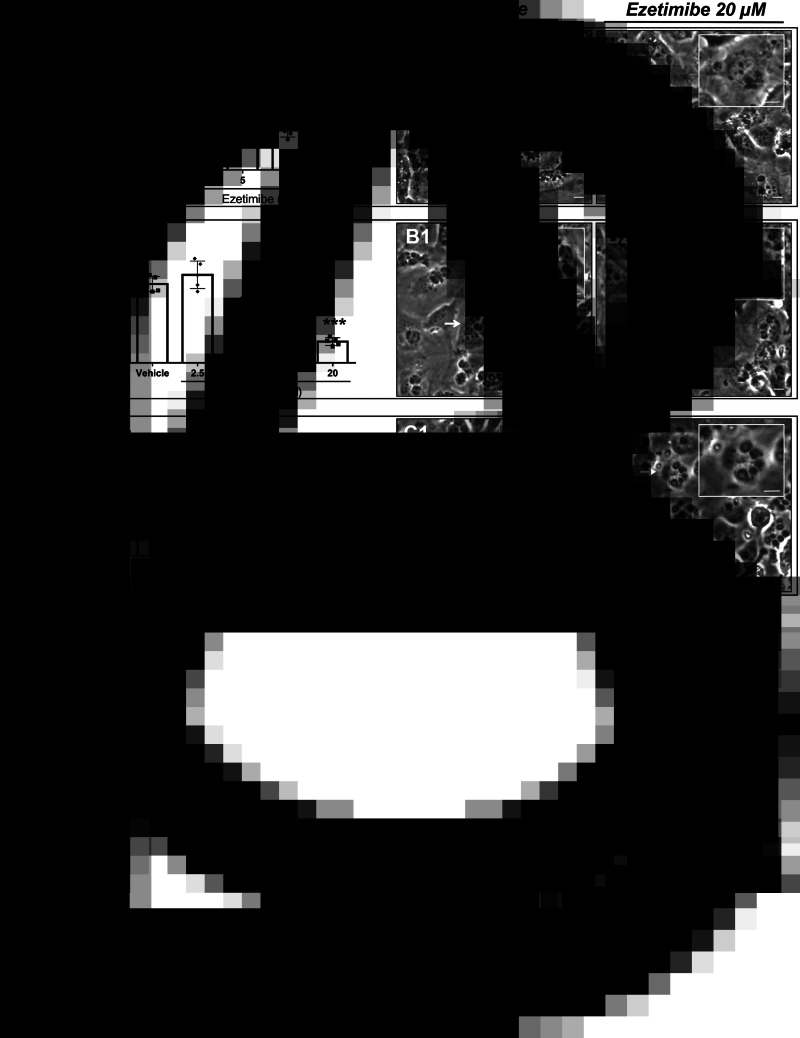
Ezetimibe treatments inhibit *T. gondii*, *N. caninum* and *B. besnoiti* tachyzoite proliferation in primary endothelial cells. BUVEC were treated with ezetimibe (2.5, 5, 10 and 20 *μ*m) 48 h before (A) *T. gondii*, (B) *N. caninum* or (C) *B. besnoiti* infection (MOI 1:5). 48 h after infection, the number of tachyzoites present in cell culture supernatants were counted (A–C). Exemplary illustration of *T. gondii* (A1−A2) *N. caninum* (B1−B2) or *B. besnoiti* (C1−C2) meront development at 24 h post infection. Scale bar represents 5 *μ*m. Bars represent means of five biological replicates ± standard deviation. * *P* ⩽ 0.05; ** *P* ⩽ 0.01; *** *P* ⩽ 0.001; **** *P* ⩽ 0.0001.

**Fig. 2.**
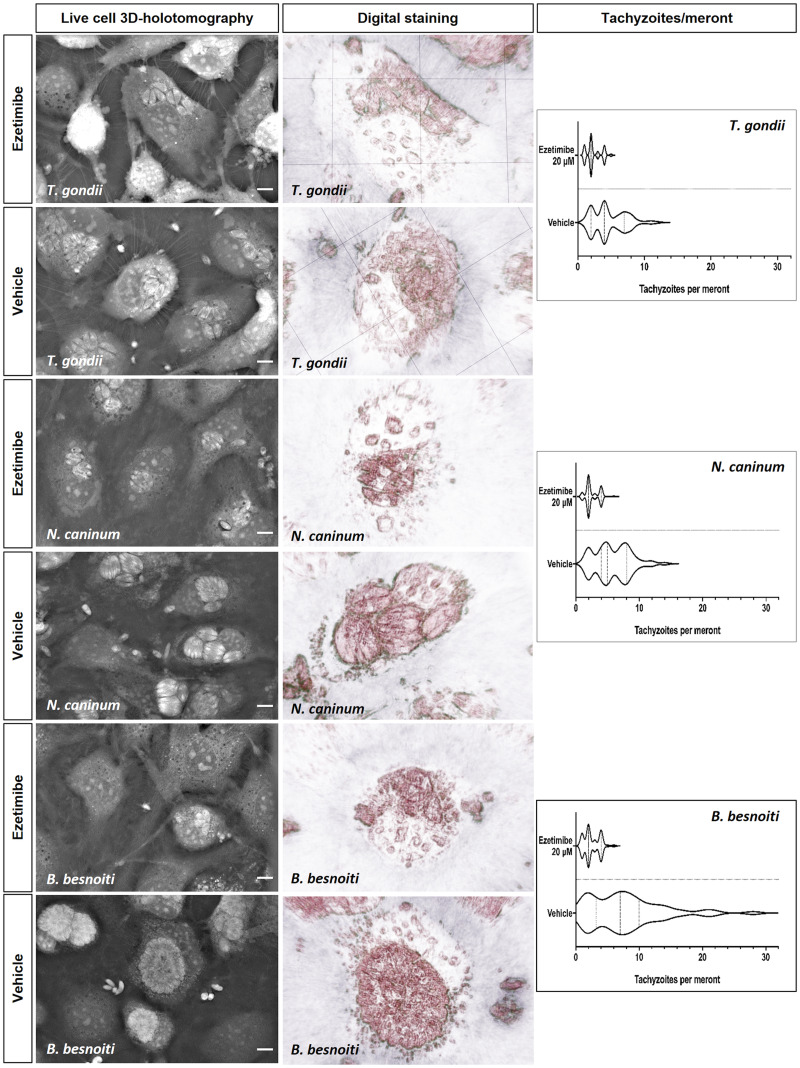
Ezetimibe treatment affects intracellular meront formation and reduces the number of *T. gondii*, *N. caninum* and *B. besnoiti* intra-meront tachyzoites. Ezetimibe-pretreated BUVEC were infected with *T. gondii*, *N. caninum* and *B. besnoiti* tachyzoites and live cell 3D holotomographic microscopy was performed at 24 h p. i. Digital staining was achieved *via* STEVE software analysis. Violin plots depict the distribution of absolute *T. gondii*, *N. caninum* and *B. besnoiti* tachyzoite number per meront.

To analyse the effects of ezetimibe on intracellular tachyzoite replication, functional inhibition experiments were performed, thereby evaluating the number of freshly released tachyzoites at 48 h p. i. from cells pre-treated and exposed to ezetimibe during the intracellular parasite proliferation stage. Overall, ezetimibe treatments significantly inhibited tachyzoite replication of *T. gondii* (10 *μ*m, *P* = 0.0397; 20 *μ*m, *P* = 0.0010; Figure 1A), *N. caninum* (20 *μ*m, *P* = 0.0078; Figure 1B) and *B. besnoiti* (10 *μ*m, *P* = 0.0059; 20 *μ*m, *P* < 0.0001; Figure 1C) in BUVEC in a dose-dependent manner. Overall, the strongest effect of ezetimibe treatments at 20 *μ*m was observed in case of *B. besnoiti* (99.2 ± 0.5% replication reduction), followed by *T. gondii* (95.7 ± 2.3% reduction) and *N. caninum* (73.1 ± 2.8% reduction). In line, phase-contrast microscopy showed an impairment in meront development for *T. gondii-* (Fig. 1A1 and 1A2), *N. caninum-* (Fig. 1B1 and 1B2) and *B. besnoiti-* (Fig. 1C1 and 1C2) infected BUVEC at 24 h p. i. To better visualize ezetimibe-based effects on parasite development, additionally live cell 3D holotomographic microscopy were performed. As illustrated in Fig. 2, treatments with ezetimibe led to reduced meront sizes in *T. gondii*, *N. caninum* and *B. besnoiti* infections (Fig. 2), without apparently affecting the morphology of non-infected host cells (data not shown). Additionally, the number of tachyzoites per PV was determined to better understand ezetimibe-derived impact on parasite development (Fig. 2). Ezetimibe treatments markedly reduced the number of tachyzoites per meront in all three parasite species (all: *P* < 0.0001), however, the strongest effect was observed for *B. besnoiti*, with a reduction of 68.2% on the mean number of tachyzoites per meront, followed by *T. gondii* and *N. caninum* showing more than 50% reduction (56.5% and 50.2%, respectively).

**Fig. 3.**
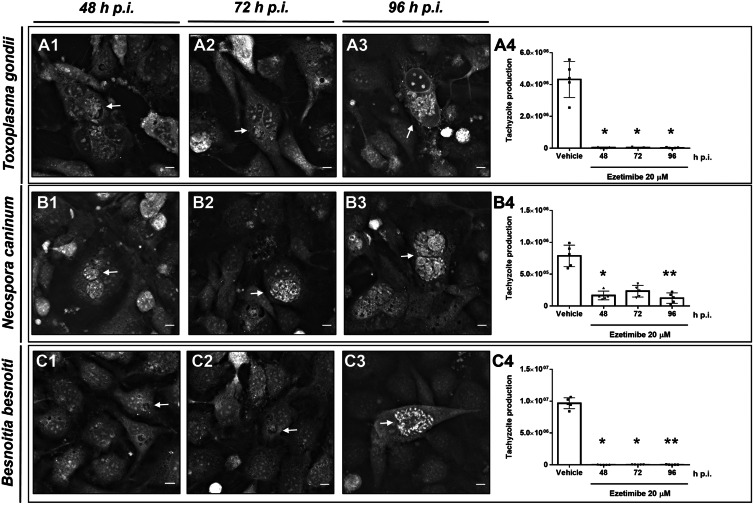
Ezetimibe blocks *T. gondii*, *N. caninum* and *B. besnoiti* tachyzoite proliferation over time. Effect of daily ezetimibe treatments on tachyzoite proliferation over time: BUVEC were treated with ezetimibe (20 *μ*m) 48 h before infection and then infected with *T. gondii* (A), *N. caninum* (B) and *B. besnoiti* (C) tachyzoites. Exemplary live cell 3D holotomographic illustration of *T. gondii* (A1−A3) *N. caninum* (B1−B3) or *B. besnoiti* (C1−C3) meront development (arrows) at 48, 72 and 96 h p. i., respectively. At 48, 72 and 96 h p. i., the number of tachyzoites present in cell culture supernatants were counted (A4, B4, C4). Bars represent means of five biological replicates ± standard deviation. * *P* ⩽ 0.05; ** *P* ⩽ 0.01; *** *P* ⩽ 0.001; **** *P* ⩽ 0.0001.

Fast replicating coccidian fulfil their replication cycle within 36–48 h p. i. in BUVEC layers *in vitro* (Taubert *et al*., 2006; Silva *et al*., 2019; Velásquez *et al*., 2019). In this context, the sustained inhibitory effect of ezetimibe over time was evaluated by counting tachyzoite production daily at 48, 72 and 92 h p. i. As depicted in Fig. 3, ezetimibe (20 *μ*m) effectively blocked *T. gondii* (99.1 ± 0.0% reduction; A1−A4), *N. caninum* (75.9 ± 7.6% reduction; B1−B4) and *B. besnoiti* (99.6 ± 0.1% reduction; C1–C4) replication over time (48, 72 and 96 h p. i.).

**Fig. 4.**
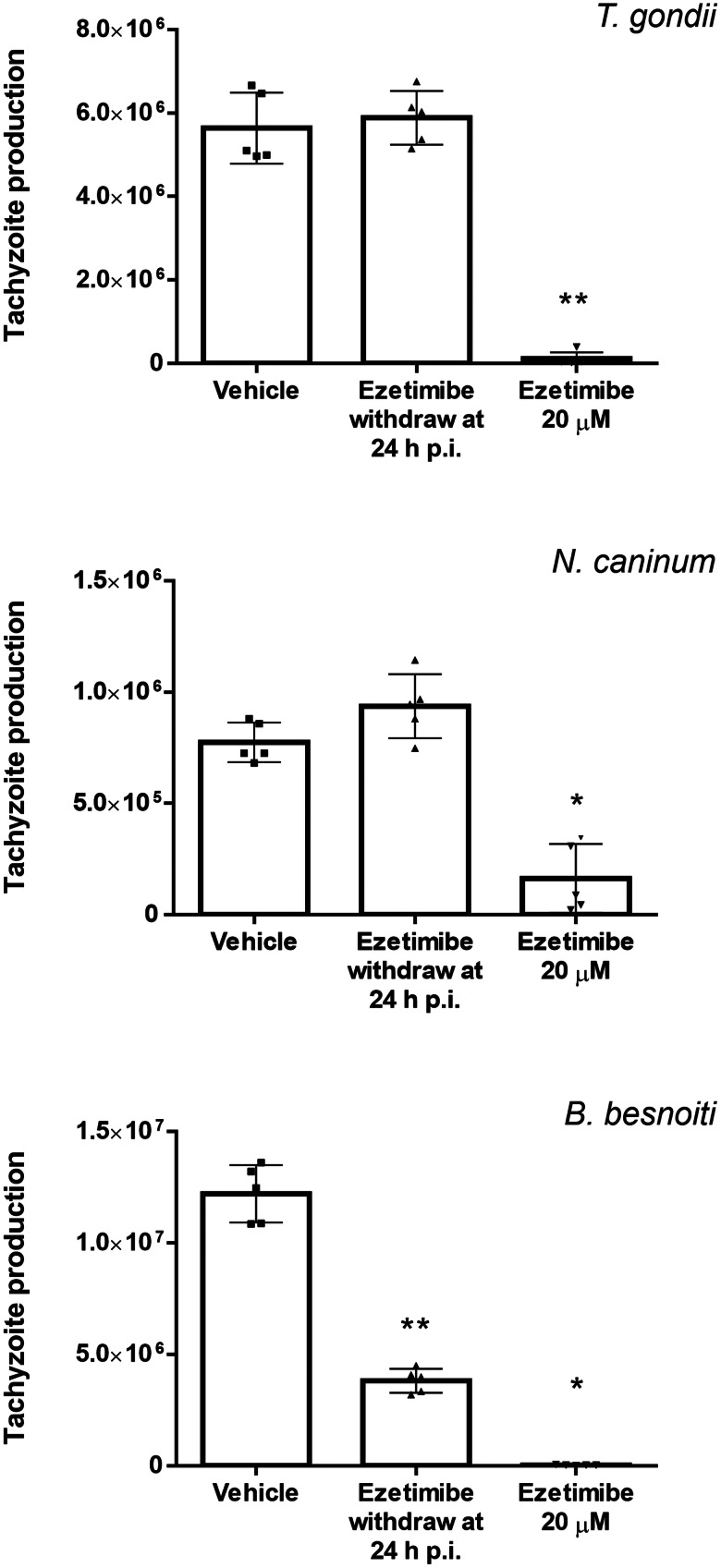
Ezetimibe withdrawal restores *T. gondii* and *N. caninum* tachyzoite replication but hardly affects *B. besnoiti* recovery. Ezetimibe-treated BUVEC were infected with *T. gondii*, *N. caninum* and *B. besnoiti* tachyzoites. At 24 h p. i., ezetimibe was removed from cultures and tachyzoites present in supernatants 24 h after withdrawal were counted. Bars represent means of five biological replicates, standard deviation. * *P* ⩽ 0.05; ** *P* ⩽ 0.01; *** *P* ⩽ 0.001; **** *P* ⩽ 0.0001.

To estimate whether ezetimibe induces either parasitostatic or parasitocidal effects, compound withdrawal experiments were performed at 24 h p. i. As illustrated in Fig. 4, remnant *T. gondii* and *N. caninum* tachyzoites quickly recovered and regained proliferative capacities 24 h after ezetimibe withdrawal. In contrast, *B. besnoiti* proved more sensitive for ezetimibe treatments showing an ongoing reduction (31.6 ± 5.3%) of tachyzoite production when compared to non-treated cells (*P* = 0.15).

### Ezetimibe treatments reduce tachyzoite infectivity but fail to affect host cell permissiveness

**Fig. 5.**
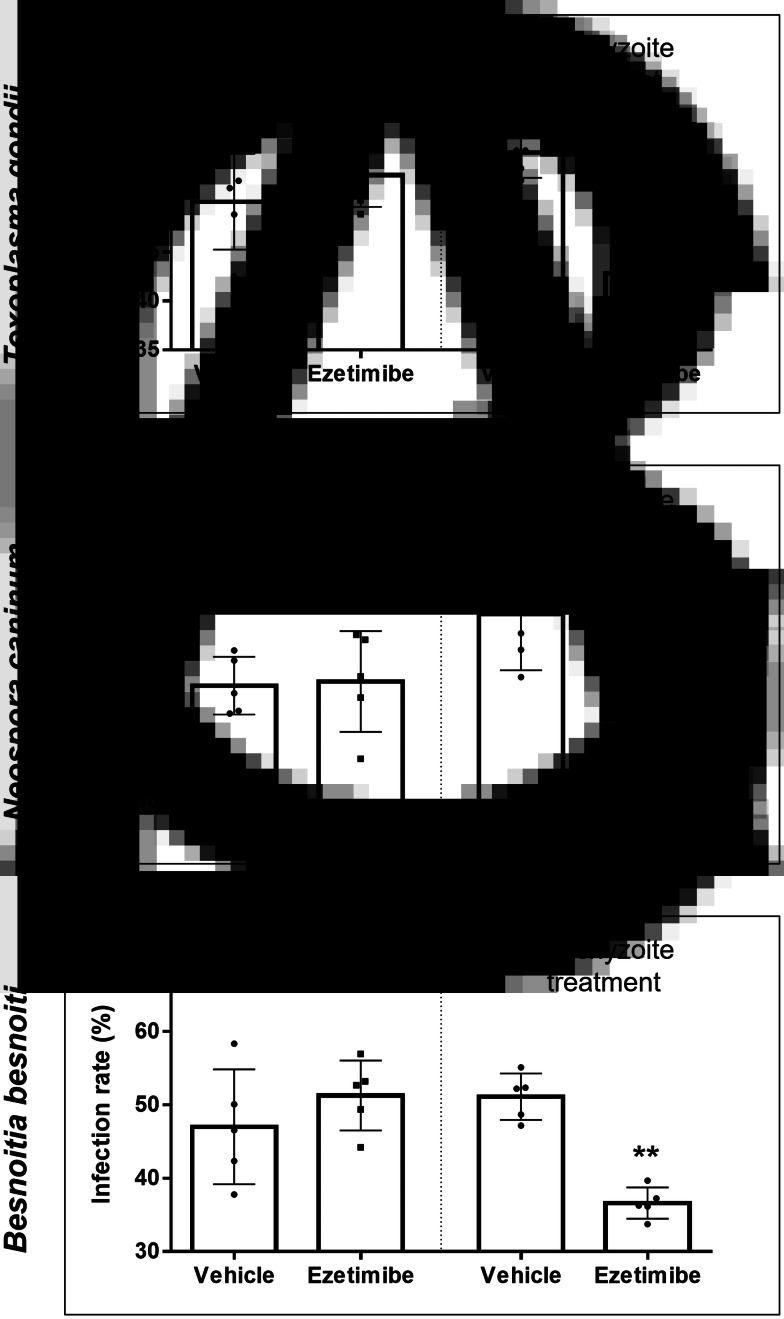
Ezetimibe treatment affects the infection capacity of *T. gondii*, *N. caninum* and *B. besnoiti* tachyzoites. Non-treated or ezetimibe-treated BUVEC were infected with ezetimibe-treated or non-treated *T. gondii* (A), *N. caninum* (B) and *B. besnoiti* (C) tachyzoites. After 4 h, infection rates were estimated. Bars represent infection rate mean of five biological replicates ± standard deviation. * *P* ⩽ 0.05; ** *P* ⩽ 0.01.

To fulfil intracellular replication tachyzoites must first actively invade the host cells. To determine if anti-parasitic effects of ezetimibe also relied on reduced infection rates, both compartments, i. e. host cells and parasites, were separately treated with ezetimibe and then tested for infection rates 4 h after infection. Therefore, BUVEC were pre-treated with ezetimibe for 48 h before infection. At 4 h p. i. non-treated control cells presented an infection rate of 50.2% (Fig. 5A), 51.8% (Fig. 5B) and 47.0% (Fig. 5C), for *T. gondii*, *N. caninum* and *B. besnoiti*, respectively. In pre-treated cells, similar infection rates were observed for each parasite species (Fig. 5A–C), thereby denying any effect of ezetimibe pre-treatments. In contrast, ezetimibe pre-treatments of fresh tachyzoites significantly reduced invasive capacities of *T. gondii* (*P* = 0.0079; Figure 5A), *N. caninum* (*P* = 0.0159; Figure 5B) and *B. besnoiti* (*P* = 0.0079; Figure 5C) tachyzoites, when compared to non-treated control stages. Here, species-dependent effects were observed since the impact of ezetimibe pre-treatments were more prominent in case of *B. besnoiti* (28.1% reduction) than in *T. gondii* (22.2% reduction) or *N. caninum* (17.3% reduction).

### Ezetimibe glucuronidation causes loss of anti-parasitic efficacy

**Fig. 6.**
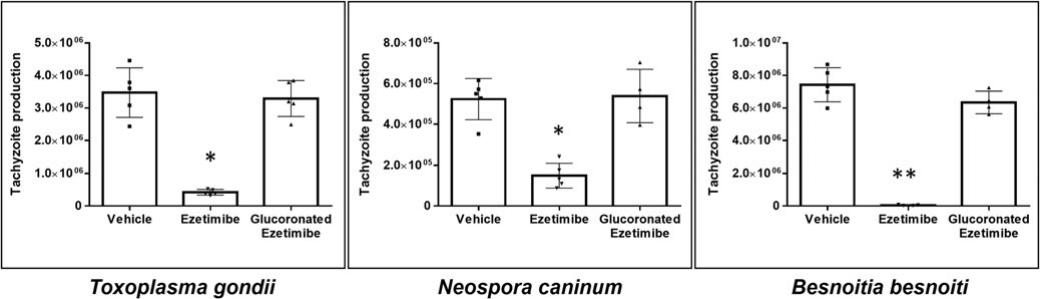
Ezetimibe-mediated anti-parasitic effects are abolished by glucoronation. Ezetimibe- or ezetimibe-glucoronide-pre-treated BUVEC were infected with *T. gondii* (A), *N. caninum* (B) and *B. besnoiti* (C) tachyzoites. 48 h after infection, the number of tachyzoites present in cell culture supernatants were counted. Bars represent means of five biological replicates ± standard deviation. * *P* ⩽ 0.05; ** *P* ⩽ 0.01; *** *P* ⩽ 0.001; **** *P* ⩽ 0.0001.

Ezetimibe-glucuronide is the major and pharmacologically active metabolite of ezetimibe following *in vivo* liver biotransformation. Thus, a functional assay to evaluate the effect of this chemically modified molecule on tachyzoite proliferation *in vitro* was performed (Fig. 6). Here, only ezetimibe but not its glucuronated derivative led to a reduction of *T. gondii*, *N. caninum* nor *B. besnoiti* tachyzoite proliferation.

### NPC1L1 gene is inconsistently transcribed in *T. gondii*-, *N. caninum*- and *B. besnoiti*-infected BUVEC

Given that NPC1L1 is described as the main target of ezetimibe in humans, the profile of gene transcription of NPC1L1 was estimated over infection kinetics (3–24 h p. i.) on *T. gondii-*, *N. caninum-* and *B. besnoiti*-infected BUVEC by qRT-PCR. Using bovine small intestine tissue samples, the functionality of the qPCR system was proved. However, infection-related data showed that neither *T. gondii-*, *N. caninum-* and *B. besnoiti*-infected BUVEC nor non-infected controls have a reliable amplification of NPC1L1 mRNAs. Specifically, as illustrated in supplementary Table 1, NPC1L1 was not detected in a consistent manner, thereby showing amplification only in some of the replicates at a rather high threshold cycle (CT > 30). Consequently, no infection-driven effect on NPC1L1 gene transcription was assumed.

### Treatments does not cause cytotoxic damage to host cells or tachyzoites

To evaluate if ezetimibe (20 *μ*) treatment induced tachyzoite dead trypan blue exclusion test was performed. Our data showed an average viability of 95.2% ± 1.3, 90.7% ± 1.9 and 93.7% ± 2.1 for *T. gondii*, *N. caninum* and *B. besnoiti* tachyzoites treated for 1 h with vehicle control (DMSO 0.06%) without significant effects provoked by ezetimibe (Fig. S1 A−C). Moreover, the cytotoxicity of ezetimibe or ezetimibe-glucuronide on endothelial host cells XTT test was performed. As illustrated in Fig. S1 D, treatments with ezetimibe or ezetimibe-glucuronide did not induce significant colorimetric changes in the formazan product compared to the vehicle control (DMSO 0.06%).

## Discussion

Cholesterol is a major component of eukaryotic cell membranes (Luo *et al*., 2020). Given that apicomplexan parasites are generally considered as defective in cholesterol synthesis, they need to obtain this molecule from the host cell. Thus, LDL-driven cholesterol uptake is considered as a key pathway to fulfil cholesterol requirements during parasite merogony in different parasite species (Labaied *et al*., 2011; Coppens, 2013; Ehrenman *et al*., 2013; Hamid *et al*., 2014; Hamid *et al*., 2015; Nolan *et al*., 2015; Taubert *et al*., 2018; Silva *et al*., 2019). Additionally, in case of *C. parvum*-infected Caco-2 cells, cholesterol is incorporated *via* NPC1L1-mediated micellar uptake (Ehrenman *et al*., 2013). NPC1L1 is a trans-membrane protein highly expressed in enterocytes that mediates sterol internalization *via* clathrin-coated vesicles and it has widely been accepted as main target of the lipid-lowering drug ezetimibe (Altmann *et al*., 2004; Garcia-Calvo *et al*., 2005; Betters and Yu, 2010).

Current data demonstrate for the first time that ezetimibe has inhibitory effects on *T. gondii*, *N. caninum*, and *B. besnoiti* tachyzoite replication in primary host endothelial cells, i.e. a host cell type that is parasitized *in vivo* during the acute phase of toxoplasmosis, neosporosis and besnoitiosis (Maley *et al*., 2003; Alvarez-Garcia *et al*., 2013; Konradt *et al*., 2016). Here, 10 *μ*m ezetimibe treatment effectively blocked *T. gondii* and *B. besnoiti* proliferation, while *N. caninum* revealed less sensitive and inhibition needed a higher concentration of 20 *μ*m. However, both concentrations are in range or even lie below the concentration known to block effectively NPC1L1 endocytosis (25–100 *μ*m, Ehrenman *et al*., 2013). Applying 20 *μ*m ezetimibe as effective concentration to all species studied, the anti-parasitic effects of this compound over time were explored. Its inhibitory effect on tachyzoite proliferation was consistent over time, since the number of newly released tachyzoites at 48, 72 and 96 h p. i. was consistently low. Thus, an overall reduction of tachyzoite production of 95.7, 73.1 and 99.2% was obtained for *T. gondii*, *N. caninum* and *B. besnoiti*, respectively. These findings in principle match data from *C. parvum*-infected Caco-2 cells (permanent cell line), where a growth reduction of 65% was achieved at 25–100 *μ*m concentrations (Ehrenman *et al*., 2013). *In vitro* anti-parasitic efficacy of ezetimibe was also reported for *L. amazonensis* where 10 *μ*m ezetimibe reduced promastigote replication, however, amastigote production was only affected at double doses (20 *μ*m; Andrade-Neto *et al*., 2016). This stage-specific discrepancy might be explained by a lower sensitivity of intracellular stages to ezetimibe treatments. Still, the concentrations here used do not necessarily support this assumption, since high effects were found at 10 *μ*m ezetimibe in case of *T. gondii* and *B. besnoiti-*infected BUVEC. Thus, stage- and species-related or even host cell type-related sensitivities may play a role. In line, *P. falciparum*-merozoite replication was effectively blocked only at much higher concentrations of 80 *μ*m ezetimibe *in vitro* (Hayakawa *et al*., 2020).

Successful parasite offspring formation relies on several defined processes starting with active cell invasion, formation of the PV, replication and egress (Black and Boothroyd, 2000). In this context, ezetimibe pre-treated extracellular tachyzoites showed impaired infection capacities, thereby hampering the replication process at the starting point. Of note, *B. besnoiti* tachyzoites appeared more sensitive to this treatment than *T. gondii* and *N. caninum* tachyzoites, the latter of which were hardly affected in their host cell invasion capacity.

A more detailed analysis of parasite intracellular development revealed an altered morphology of meronts in the case of all three parasites in treated host cells, suggesting that prolonged ezetimibe exposition indeed affected tachyzoite physiology, thereby reducing or hampering their ability to proliferate. Residual effects of ezetimibe on *in vitro* tachyzoite replication were evaluated by drug-withdrawal experiments. Notably, *T. gondii* and *N. caninum* tachyzoites recovered within 24 h post withdrawal and proliferated at normal replication rates thereby showing that ezetimibe mainly induced developmental arrest but did not kill the parasites. In contrast, *B. besnoit* tachyzoites suffered more profoundly from ezetimibe treatments since drug removal did not result in full recovery of parasite replication. These reactions indeed indicated species-specific sensitivity towards ezetimibe.

Overall, it is challenging to dissect if ezetimibe exclusively affects the parasites and/or the host cells, or if cumulative effects are to be considered. However, the current data showed that pre-treatments of host cells did not affect infection permissiveness since infection rates were similar in treated and control cells, when using non-treated tachyzoites for infection. Nevertheless, ezetimibe treatment of tachyzoites before host cell invasion led to a significant reduction of infection rates, suggesting that ezetimibe also directly acts on tachyzoite invasive capacity in a rather species-dependent manner. Besides this mode of action, anti-parasitic activity of ezetimibe was also associated with inhibition of parasite replication within PV. By using live cell 3D holotomographic microscopy as a reliable tool for 3D cell visualization *in vivo* (Silva *et al*., 2019; Velásquez *et al*., 2019), we showed that ezetimibe-treated host cells infected with *T. gondii*, *N. caninum* and *B. besnoiti* presented a reduced number of tachyzoites per meront. Summarizing these data, ezetimibe might act on both, extra- and intracellular tachyzoites.

Hypolipidaemic/cholesterol-lowering properties of ezetimibe have previously been reported for humans as well as animals (Bays *et al*., 2001; van Heek *et al*., 2001; Knopp *et al*., 2003). *In vivo,* this compound undergoes phase II metabolism to form a glucuronide conjugate, thereby improving NPC1L1-specific affinity and binding capacities (Garcia-Calvo *et al*., 2005). Besides being the major metabolite detected in plasma (Garcia-Calvo *et al*., 2005), ezetimibe-glucuronide is therefore considered as main active metabolite in ezetimibe treatments *in vivo*. To parallel *in vivo* situation, additional studies on the effect of ezetimibe-glucuronide on *T. gondii, B. besnoiti* and *N. caninum* tachyzoite proliferation *in vitro* were performed. Unexpectedly, treatments with ezetimibe-glucuronide failed to hamper intracellular tachyzoite replication thereby implicating that ezetimibe-mediated anti-parasitic effects might be NPC1L1-independent. In line, we were not able to demonstrate a consistent infection-driven induction of NPC1L1 mRNAs since these gene transcripts could hardly be detected in infected BUVEC or control cells even though intestinal control tissues gave good PCR signals. Consequently, we here assume a very low expression of this transporter in BUVEC. Likewise, the presence of NPC1L1 protein expression by Western blotting was not achieved, so far (unpublished data). Noteworthy, ezetimibe was originally identified as an ACAT II inhibitor (Clader, 2004) and therefore as acting on other potential targets besides NPC1L1. Irrespective of this, it is well documented that NPC1L1 incorporates cholesterol through an ezetimibe-sensitive pathway, however, the binding mechanism between ezetimibe and NPC1L1 remains unknown (Betters and Yu, 2010). Recently, it has been reported that ezetimibe, but not ezetimibe-glucuronide, reduces the cellular content of cholesteryl esters in a NPC1L1-independent manner in human monocytes, implying an inhibition of ACAT II (Orso *et al*., 2019). Likewise, we here propose that the current ezetimibe-mediated anti-coccidian effects may rather be linked to an inhibition of cholesterol esterification. In agreement, the importance of functional cholesterol esterification for coccidian replication was already confirmed for *T. gondii* (Sonda *et al*., 2001), *B. besnoiti* (Silva *et al*., 2019) and *E. bovis* (Hamid *et al*., 2014). However, the actual role of ezetimibe in cholesterol esterification and its cytosolic targets in primary bovine endothelial host cells should be further addressed in future studies. Even though *in vitro* studies have been published reporting anti-parasitic activities of ezetimibe treatments, *in vivo* evidence still needs to be addressed. Nevertheless, administration of ezetimibe to *L. amazonensis*-infected mice led to reduced parasite burden and boosted anti-leishmanial activity of ketoconazole (Andrade-Neto *et al*., 2016). However, given that ezetimibe treatments of *P. yoelii*-infected mice failed to affect parasitaemia (Kume *et al*., 2016) phylum-derived differences have to be assumed.

In conclusion, the current study shows that ezetimibe effectively inhibits *T. gondii*, *N. caninum* and *B. besnoiti* tachyzoite replication in BUVEC in a time-sustained but reversible manner. Apparently, the anti-coccidian effect of ezetimibe is associated with both impairment of tachyzoite infectivity and intracellular replication blockage. Of note, we additionally observed that ezetimibe-glucuronide does not interfere with parasite replication thereby suggesting an NPC1L1-independent anti-parasitic mechanism.

## Data Availability

All data are available in the manuscript and Supplementary data files.

## Supplementary material

For supplementary material accompanying this paper visit https://doi.org/10.1017/S0031182021000822. click here to view supplementary material
