# Supplementary material for: Ezetimibe blocks Toxoplasma gondii-, Neospora caninum- and Besnoitia besnoiti-tachyzoite infectivity and replication in primary bovine endothelial host cells
Source: Parasitology. 2021 May 24;148(9):1107–15. doi: 10.1017/S0031182021000822 (PMC8273898; doi:10.1017/S0031182021000822)
Supplement: Supplementary file 1 [file S0031182021000822sup001.docx]

**Supplementary data**

**
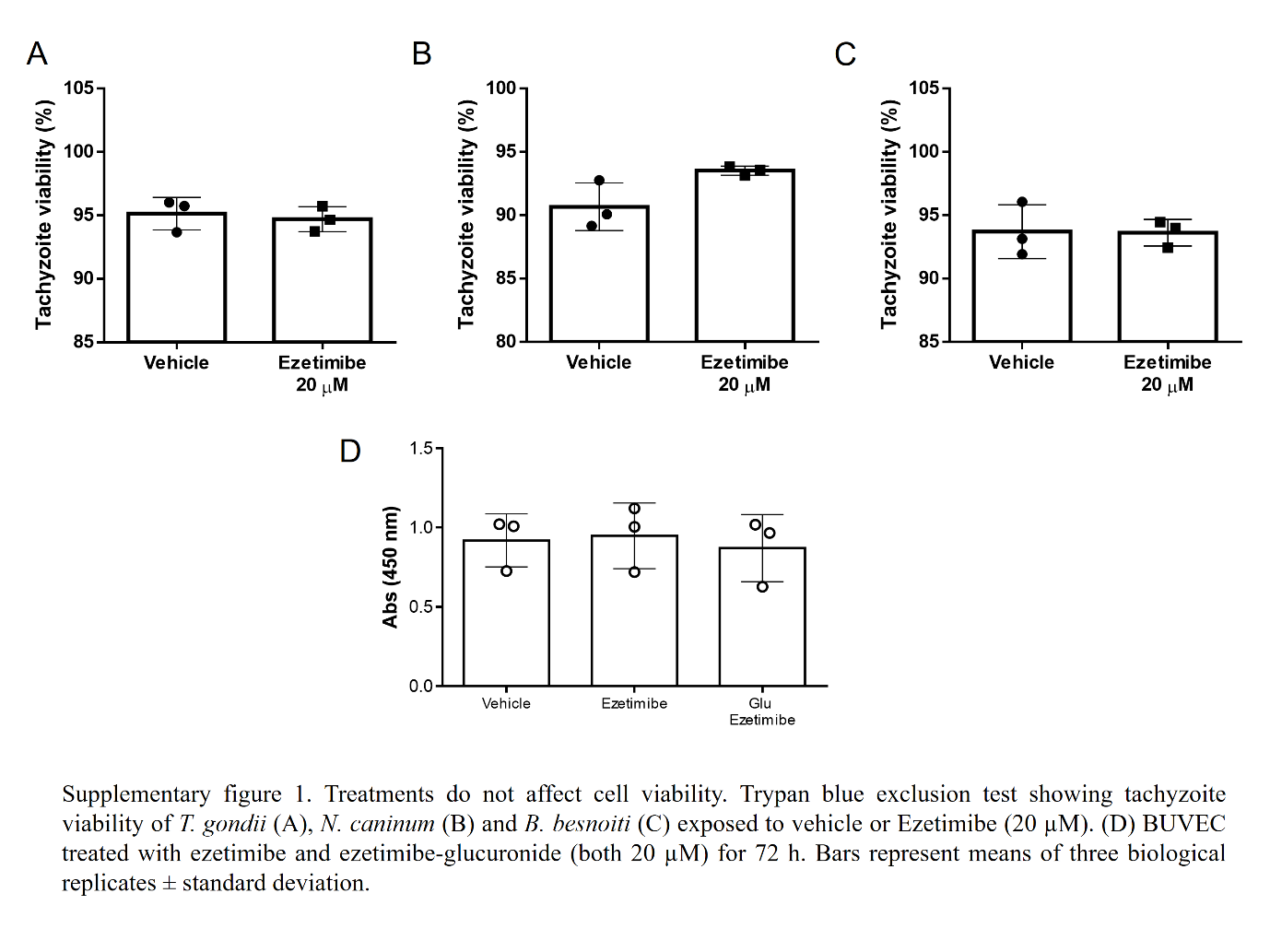
**

**Supplementary figure 1. Ezetimibe and ezetimibe-glucuronide treatments do not affect BUVEC viability.** DMSO, ezetimibe- or ezetimibe-glucuronide-pre-treated BUVEC were treated for 72 h and then incubated 4h with XTT working solution. Bars represent means of three biological replicates ± standard deviation.

**Supplementary table 1.** Representative cycle threshold for NPC1L1 and GAPDH in non- and 6 h p.i. infected BUVEC. Bovine small intestine tissue were used as positive control.

|  | CT NPC1L1 | | CT GAPDH |  |  |  |  |  |
| --- | --- | --- | --- | --- | --- | --- | --- | --- |
| Bovine small intestine | 25,24 | | 19,95 |  |  |  |  |  |
|  | 24,79 | | 20,01 |  |  |  |  |  |
|  |  | | |  |  |  |  |  |
|  |  | | CT NPC1L1 | CT GAPDH |  |  | CT NPC1L1 | CT GAPDH |
| BUVEC isolate 1 | n.i. | | n.d. | 17,85 | BUVEC isolate 2 | n.i. | 37,87 | 18,34 |
|  |  |  | n.d. | 17,55 |  |  | n.d. | 18,19 |
|  | *T. gondii* 6 h p.i. | | n.d. | 19,96 |  | T. gondii 6 h p.i. | 38,98 | 17,86 |
|  |  |  | 37,35 | 17,45 |  |  | 37,63 | 18,22 |
|  | *N. caninum* 6 h p.i. | | n.d. | 18,75 |  | N. caninum 6 h p.i. | 37,08 | 18,53 |
|  |  |  | n.d. | 19,13 |  |  | 37,11 | 18,59 |
|  | *B. besnoiti* 6 h p.i. | | 37,74 | 19,03 |  | B. besnoiti 6 h p.i. | n.d. | 19,11 |
|  |  |  | 34,94 | 19,03 |  |  | n.d. | 19,04 |
|  |  | |  |  |  |  |  |  |
|  | |  | CT NPC1L1 | CT GAPDH |  |  | CT NPC1L1 | CT GAPDH |
| BUVEC isolate 3 | | n.i. | n.d. | 16,18 | BUVEC isolate 4 | n.i. | 37,26 | 16,35 |
|  |  |  | 37,35 | 16,6 |  |  | n.d. | 16,26 |
|  |  | T. gondii 6 h p.i. | 36,96 | 18,34 |  | T. gondii 6 h p.i. | 36,19 | 18,41 |
|  |  |  | n.d. | 18,38 |  |  | 36,28 | 18,28 |
|  |  | N. caninum 6 h p.i. | n.d. | 18,55 |  | N. caninum 6 h p.i. | n.d. | 19,07 |
|  |  |  | n.d. | 18,58 |  |  | 38,24 | 18,59 |
|  |  | B. besnoiti 6 h p.i. | 36,91 | 18,57 |  | B. besnoiti 6 h p.i. | 34,76 | 19 |
|  |  |  | 36,67 | 19,04 |  |  | 33,85 | 19,1 |
|  | |  | CT NPC1L1 | CT GAPDH |  |  |  |  |
| BUVEC isolate 5 | | n.i. | n.d. | 18,37 |  |  |  |  |
|  |  |  | n.d. | 18,36 |  |  |  |  |
|  |  | T. gondii 6 h p.i. | n.d. | 18,38 |  |  |  |  |
|  |  |  | 37,18 | 18,46 |  |  |  |  |
|  |  | N. caninum 6 h p.i. | n.d. | 17,66 |  |  |  |  |
|  |  |  | n.d. | 17,67 |  |  |  |  |
|  |  | B. besnoiti 6 h p.i. | 33,71 | 18,59 |  |  |  |  |
|  |  |  | 33,94 | 18,28 |  |  |  |  |
